# Lead exposure across early life in Latin America and the Caribbean: prevention strategies and reproductive health considerations

**DOI:** 10.3389/frph.2026.1761778

**Published:** 2026-03-18

**Authors:** Maíra Boda, Elizeu Chiodi Pereira, Eliel Lucas de Sousa Capaz Lima, Kelly Polido Kaneshiro Olympio

**Affiliations:** 1 Departamento de Saúde Ambiental, Faculdade de Saúde Pública, Universidade de São Paulo, São Paulo, Brazil; 2 Expossoma e Saúde do Trabalhador (Exposome and Worker’s Health - eXsat), Faculdade de Saúde Pública, Universidade de São Paulo, São Paulo, Brazil

**Keywords:** blood, Caribbean, children, Latin America, lead, public policies, reproductive health

## Abstract

Lead exposure remains a persistent public health problem, with direct implications for reproductive and child health. Toxic and bioaccumulative, the metal persists in the environment due to historical use in fuels, paints, and ceramics, as well as industrial and mining activities. The aim of this study was to synthesize recent evidence on lead exposure in children from Latin America and the Caribbean (LAC), assessing outcomes related to human reproduction. A literature review was conducted according to PRISMA guidelines on the PubMed, Web of Science, and LILACS databases, covering the period from January 1, 2022, to January 30, 2026. The search was carried out on January 30, 2026, and only original articles in English, Portuguese, and Spanish were included. 165 studies were identified, of which 22 met the inclusion criteria. Four studies evaluated maternal exposure during pregnancy, while the remaining publications addressed exposures during childhood or in the general population. Some children participating in the included studies had blood lead levels exceeding the international reference value of 3.5 μg·dL^−^^1^. Glazed ceramics, mining activities, improper e-waste management, and water and soil contamination emerged as the main exposure sources. Negative effects included cognitive deficits, learning difficulties, behavioral changes, and, in some contexts, juvenile delinquency. Despite regulatory advances and updated reference values, shortcomings remain, such as a lack of systematic biomonitoring and longitudinal studies. Reproductive effects beyond childhood remain, such as premature births and low birth weight. With no safe level, defining priorities for reproductive, child, and intergenerational health in LAC is paramount.

## 1 Introduction

Lead is a toxic metal, and human exposure is considered one of the most persistent and serious public health problems globally, with direct implications for child and reproductive health. For women of reproductive age, lead affects the body through premature birth, spontaneous abortions (1), infertility (2), pre-eclampsia (3), and increased Blood Lead Levels (BLL) during menstruation due to bone lead mobilization (4). In men, exposure reduces sperm concentration, motility, morphology and fertility (5, 6).

Reproductive-age women, especially during pregnancy or lactation, are highly vulnerable to lead due to physiological, environmental and social factors (7). Lead accumulates in bone (8) and is released during pregnancy, crossing the placenta and exposing the fetus during critical development, reflecting intergenerational exposure.

Although no safe BLL has been identified (9, 10), public health agencies use reference values to guide surveillance and interventions. The Centers for Disease Control and Prevention (CDC) recommends that BLL during pregnancy not exceed 5 µg·dL^−^^1^, with regular monitoring if exceeded (8). For children, the CDC updated the blood lead reference value from 5.0 to 3.5 µg·dL^−^^1^ as a surveillance benchmark, not a toxicity threshold, to identify children with higher BLL than peers (11).

In early childhood, lead exposure affects physiological and behavioral development of the nervous (12) and gastrointestinal (8) systems. Exploratory behaviors increase ingestion of lead-contaminated dust and soil (13). Neurotoxicity is the main concern, with strong evidence linking lead exposure to cognitive deficits (8, 14), lower school performance, learning difficulties (15, 16), attention problems (16), hyperactivity (9), and antisocial behaviors (9, 14).

At higher exposure levels, lead toxicity presents as overt clinical disease. Classical lead poisoning (saturnism) causes severe outcomes (abdominal pain, seizures, neurological impairment, and coma), typically at markedly elevated BLL. In clinical practice, BLLs ≥45 µg·dL^−^^1^ indicate severe poisoning requiring immediate intervention, including chelation therapy (11, 17). While high-dose effects are well established, chronic low-level exposure, often asymptomatic, is increasingly recognized for causing long-term developmental and neurological harm, particularly in children (17).

Therefore, this work synthesizes available scientific evidence on the impacts of lead exposure on reproductive health and child development in LAC. The findings can inform stakeholder decision-making on lead toxicity from pregnancy through adolescence, reinforcing its ongoing public health relevance.

## 2 Methodology

A literature review was conducted following the Preferred Reporting Items for Systematic Reviews and Meta-Analyses (PRISMA) methodology (18), covering January 1, 2022, to January 30, 2026, in PubMed, Lilacs, and Web of Science, using descriptors including “Lead/blood,” “Child,” and “Latin America OR Caribbean Region”. Searches were performed on January 30, 2026. Strategies are detailed in Supplementary Table S1.

Only original studies of mother–child dyads or children aged 0–18 years living in LAC, published in English, Portuguese, or Spanish, that reported capillary or venous BLL biomonitoring were included. Theses, dissertations, reviews, and commentaries were excluded.

Two authors (MB, ELSCL) performed blinded selection of retrieved studies, applying pre-established inclusion and exclusion criteria. Disagreements were resolved by a third reviewer (ECP). Authors of included studies were contacted when additional data were needed. The review was conducted using the Rayyan platform (19) and registered in the PROSPERO platform (CRD420251086114).

## 3 Results

### 3.1 Overview

Of 165 studies identified, 99 were excluded for not meeting the inclusion criteria, leaving 22 included in the review. Results are summarized in Table 1, and the selection flowchart appears in the Supplementary Material S1.

**Table 1:** Studies describing BLLs in Latin American mothers and children, or in children only, published between January-2022 to January-2026.

Country (city, state)	Number (*N*) and age of children (year of blood collection)	Descriptive characteristics of exposure or non-exposure	Sample type	Geometric mean (GM) or Arithmetic mean (AM) or Median of BLL (IC 95% or range, or SD)	Bibliographic reference
Argentina *(La Plata)*	*N* = 131 1–6 years (2014–2015)	Cross-sectional study. Children living in La Plata and suburban areas near major industrial oil refinery sites in South America.	Venous blood	GM: 1.90 µg.dL^−1^	Disalvo et al. (2022) (27)
Argentina *(La Plata)*	*N* = 392 1–6 years (2012–2017)	Cross-sectional study. Exposure defined as living <100 m from gas stations, bus stops, workshops, high-traffic roads, dumpsites, or polluted streams. BLL >5 µg/dL considered elevated.	Venous blood	Overall GM: 1.96 µg.dL^−1^; GM in children >2 years: 2.01 µg.dL^−1^	Disalvo et al. (2025) (28)
Brasil *(Aratuipe)*	*N* = 143 5–13 years (2021)	Cross-sectional study. RG: reference group; EG: exposed group (residents of a traditional lead-glazed pottery community).	Whole blood	Median: 1.0 µg.dL^−1^ EG: 2.3 µg.dL^−1^ RG: 0.1 µg.dL^−1^	Bah et al. (2022) (13)
Chile *(Arica)*	*N* = 668 0–11 years (2016–2021)	Cross-sectional study. Exposure defined as living ≥6 months in a mining waste disposal area; maternal residence ≥3 months during pregnancy also considered exposure.	Blood	Median BLL: 1.9 µg.dL^−1^	Medel-Jara et al. (2023) (26)
Jamaica *(Kingston)*	*N* = 688 2–8 years (2009–2012)	Case-control study. Children with autism spectrum disorder and typical development; environmental exposure and diet.	Venous blood	GM: 1.74 µg.dL^−1^ Controls: 2.27 µg.dL^−1^	Rahbar et al. (2014) (37)
México *(Ciudad de Mexico)*	*N* = 549 6–7 years (2014–2018)	Prospective cohort study. Lead exposure from continuous use of lead-glazed ceramic cookware.	Blood	Mean: 2.4 ± 2.6 µg.dL^−1^	Merced-Nieves et al. (2022) (31)
México *(General)*	*N* = 1158 1–4 years (2022)	Cross-sectional study. Lead exposure through the use of lead-glazed pottery, environmental exposure, and para-occupational* exposure.	Capillary blood	GM: 3.45 µg.dL^−1^	Bautista-Arredondo et al. (2023) (22)
Mexico *(General)*	*N* = 1394 1–5 years (2018)	Cross-sectional study. Lead exposure in rural areas and small towns (<100,000 inhabitants).	Capillary blood	AM: 5.49 µg.dL^−1^ (range: 3.3–47)	Córdoba-Gamboa et al. (2023) (20)
Mexico *(Ciudad de Mexico)*	*N* = 533 4 years (2007–2011)	Prospective cohort study. Prenatal and early childhood lead exposure.	Venous blood	Median: 1.71 µg.dL^−1^	Liu et al. (2023) (32)
Mexico *(Gereral)*	*N* = 3,127 1–4 years (2018–2019)	Cross-sectional study. Lead poisoning is defined as BLLs equal to or higher than 5 µg/dL.	Capillary blood	GM: 3.6 µg.dL^−1^; GM for children with BLL ≥5 µg. dL^−1^: 7.34 µg.dL^−1^.	Figueroa et al. (2024) (15)
Mexico *(Ciudad de Mexico)*	*N* = 704 (1994–2008) 0–5 years *N* = 595 (2008–2012) 6–16 years	Prospective cohort study. childhood lead exposure, from the prenatal period through adolescence.	Venous blood	Median (1994–2008): 5,19 µg.dL^−1^ Median (2008–2012): 2,62 µg.dL^−1^	Reyes Sánchez et al. (2022) (35)
Mexico *(Coahuila)*	*N* = 34,621 0–15 years (2010–2022)	Epidemiological Surveillance Study. Children and adolescents living in residential neighborhoods located within a 2 km radius of a metallurgical industry	Venous blood	Median: 3.61 µg.dL^−1^ (1.18–10.3 µg.dL^−1^)	Ríos-Sánchez et al. (2025) (23)
Mexico *(Ciudad de Mexico)*	*N* = 576 6–8 years (2007–2017)	Prospective cohort study. Prenatal and early childhood lead exposure.	Blood	Median (second trimester mothers): 2.9 µg.dL^−1^ Median (third trimester mothers): 3.1 µg.dL^−1^ Median in children: 1.7 µg.dL^−1^	Svensson et al. (2025) (33)
Mexico *(Ciudad de Mexico)*	*N* = 506 (1997–2003) 1–2 years	Prospective cohort study. Prenatal and early childhood lead exposure	Venous blood	Mean (third trimester mothers):5.5 ± 4.1 µg.dL^−1^ Mean in children (12 months):4.5 ± 3.1 µg.dL^−1^ Mean in children (24 months): 4.7 ± 3.5 µg.dL^−1^	Tagelsir et al. (2023) (34)
Mexico *(Gereral)*	*N* = 774 children 1–4 years (2022–2023)	Cross-sectional study. Three groups of exposure sources were analyzed: use of glazed clay, environmental exposure, and para-occupational* exposure.	Capillary blood	GM: 3.62 µg.dL^−1^	Téllez-Rojo MM et al. (2024) (21)
Mexico *(Ciudad de Mexico)*	*N* = 602 6–9 years (2007–2011)	Prospective cohort study. Environmental exposure in large urban centers	Venous blood	Mean: 2.36 ± 2.31 µg.dL^−1^	Lane et al. (2025) (36)
Peru *(Ica)*	*N* = 40 <1 year (2019)	Exploratory and cross-sectional study. Lead exposure through maternal diet, water, air, or soil ingestion, reaching infants via breast milk and later complementary feeding.	Venous blood	Mean: 2.05 ± 1.35 µg.dL^−1^	Linares et al. (2024) (25)
Uruguai *(Montevideu)*	*N* = 759 7 years (2009–2019)	Cross-sectional study. Children from schools with reported lead exposure; possible occupational exposure among caregivers was also evaluated.	Venous blood	2009: Mean: 4.8 ± 2.6 µg.dL^−1^; 2019: Mean: 1.4 ± 1.4 µg.dL^−1^.	Queirolo et al. (2023) (38)
Uruguai *(Montevideu)*	*N* = 252 5–9 years (2009 -2013)	Cross-sectional study. Identified sources: metallurgical industries, battery recycling, lead wire and pipe factories, old paint, dust, and shoes.	Venous blood	Mean: 4.0 ± 2.2 µg.dL^−1^	Agudelo et al. (2024) (16)
Uruguai *(Montevideu)*	*N* = 245 5–9 years (2009–2013)	Cross-sectional study. Children from low-income schools located in areas at risk for metal exposure.	Venous blood	Mean: 4.0 ± 2.2 µg.dL^−1^.	Agudelo et al. (2024) (24)
Uruguai *(Montevideu)*	*N* = 303 (2009–2013) *N* = 443 (2015–2019) 6–7 years	Exploratory and cross-sectional study. Lead exposure through maternal diet, water, air, or soil ingestion, transmitted to infants via breast milk and later by complementary feeding. Phase I: included only private-school children. Phase II: included both private and public-school children.	Venous blood	Median in Phase I: 3.8 µg.dL^−1^ Median in Phase II: 1.3 µg.dL^−1^	Kordas et al. (2024) (30)
Uruguai (*Montevideu)*	*N* = 38 9–13 years (2012–2019)	Cohort study. Children exposed to lead, even at low levels, in household environments	Venous blood	Mean: 1.46 ± 1.22 µg.dL^−1^	Barg et al. (2025) (29)

**Figure 1:**
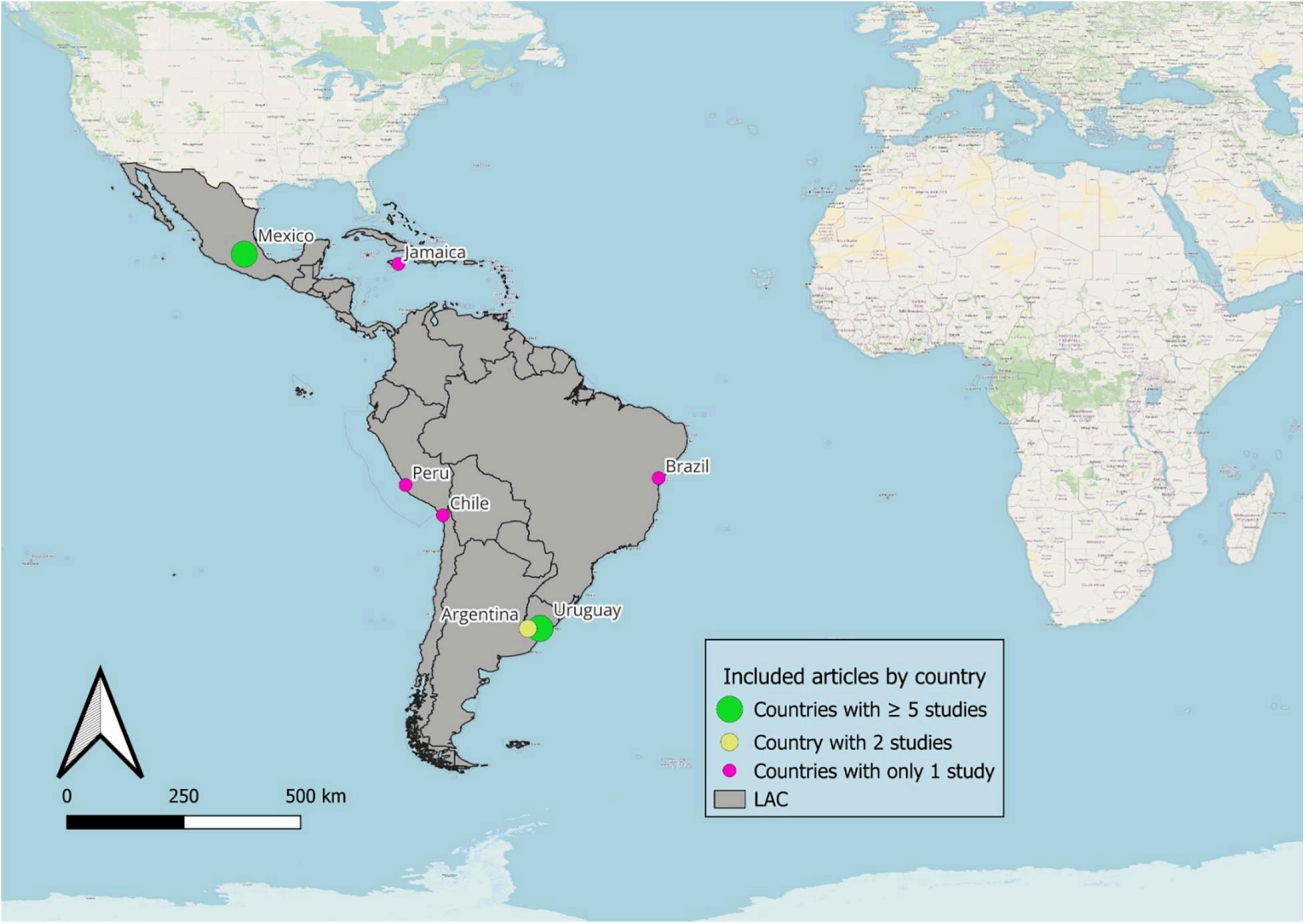
Distribution of included studies by country.

Mexico accounted for the largest share of included studies on childhood and reproductive/pregnancy lead exposure (*n* = 11; 50%), followed by Uruguay (*n* = 5; 22.7%) and Argentina (*n* = 2; 9.1%). The remaining studies were conducted in other LAC countries, including Brazil, Chile, Peru, and Jamaica, each contributing one study (*n* = 1; 4.5%). Four studies examined maternal exposure during pregnancy and its effects on child health. The geographic distribution of included studies is shown in Figure 1.

### 3.2 Sources of lead exposure

Multiple lead exposure sources were identified. In Mexico, a national survey showed that glazed ceramics with lead-based enamel remain one of the main sources of exposure (20). Similarly, in Brazil, this sources was also identified in communities where artisanal production of ceramic utensils remains part of cultural practice (13).

Téllez-Rojo et al. (21) and Bautista-Arredondo et al. (22) reported para-occupational exposure from family members working with lead sources such as industrial activities, metal processing, recycling, and e-waste, which remain important exposure routes alongside glazed ceramics in Mexico. Additionally, Ríos-Sánchez et al. (23) conducted a longitudinal epidemiological surveillance study among children and adolescents living within 2 km of a large metallurgical smelter and reported a decline in BLL between 2010 and 2022, although median concentrations remained elevated, decreasing from 5.1 to 3.3 µg.dL^−1^ between 2010 and 2022, with the highest levels observed in 1-year-old children (median: 5.5 µg.dL^−1^). Studies in Uruguay reported concentrations of about 2.0 µg.dL^−1^ between 2015 and 2019 among children living near metallurgical and metal-waste industries. Agudelo et al. (24) found an average BLL of 4.0 µg.dL^−1^, linked to environmental contamination from industrial waste, exceeding CDC reference values (3.5 µg.dL^−1^) (11).

In Peru, Linares et al. (25) found median BLL of 0.026 µg.dL^−1^ in breast milk from mothers in agricultural/mining areas, with most samples above reference limits. In Arica, Chile, an industrially contaminated area, median BLL was 1.9 µg.dL^−1^ (26). In La Plata, Argentina, near a major oil refinery, BLL averaged 1.9 µg.dL^−1^ (1.71–2.10) (27).

Contaminated water, soil, and food are major sources of childhood lead exposure, through ingestion or inhalation (28). In Uruguay, 65.7% of children consumed unfiltered tap water (29), and Kordas et al. (30) highlighted the influence of diet quality during early school years.

### 3.3 Lead exposure and effects on reproductive and child health

Regarding mother–child studies, in Mexico, Merced-Nieves et al. (31) associated prenatal lead exposure with poorer behavioral performance, with girls showing slower learning and attention responses and boys exhibiting greater difficulties with time control and response consistency. Liu et al. (32) showed that prenatal and preschool lead exposure impairs inhibitory control and, based on umbilical cord blood, confirmed placental transfer, highlighting risks for reproductive and child health.

A recent Mexican mother–child study found that children aged 6–8 years (median BLL 1.7 µg.dL^−1^) had higher forgetting rates linked to childhood lead exposure, whereas prenatal exposure was not significant (33). Another Mexican study on molar hypomineralization reported maternal mean BLLs of 5.1–5.7 µg.dL^−1^during pregnancy and child BLLs of 4.5 µg.dL^−1^ (12 months) and 4.7 µg.dL^−1^ (24 months). Prenatal exposure, especially in the third trimester, was significantly associated with the outcome, while postnatal BLLs were not (34). A life-course study found prenatal lead exposure (measured in maternal bone) significantly associated with adolescent conduct problems and aggressiveness, whereas BLL in childhood and adolescence were not significantly associated with these outcomes (35).

Regarding childhood effects, studies have associated elevated BLL with cognitive deficits, behavioral changes, and ADHD symptoms (29). Agudelo et al. (16, 24) found that higher BLL correlated with lower math skills and poorer vocabulary and language development in schoolchildren. In Mexico, lead exposure was associated with an estimated US$ 33.01 billion annual productivity loss from IQ decline in children under five (15). Córdoba-Gamboa et al. (19) linked lead and malnutrition to impaired language development. Lane et al. (36) reported cognitive impacts in children with a mean BLL of 2.36 ± 2.31 µg.dL^−1^. In Brazil, Bah et al. (13) found that children living near ceramic production facilities had twice the BLL of controls, with no significant IQ decline. In Argentina, a study assessed environmental risk factors within 100 m of residences, including gas stations, workshops, blacksmiths, bus stops, and busy streets. The geometric mean BLL was 1.96 µg.dL^−1^ (28), with hematological changes, including anemia. Disalvo et al. (27) reported a BLL of 1.90 µg.dL^−1^, that, despite being below reference limits, induced lipid peroxidation and potential neurobehavioral effects. In Jamaica, prenatal and childhood lead exposure was associated with epigenetic changes (37), while in Peru, Linares et al. (25) reported a mean BLL of 2.05 ± 1.35 µg.dL^−1^ in children under one year old.

Lastly, in Uruguay, BLL around 2.0 µg.dL^−1^ were associated with deficits in cognitive control and behavior (38). Kordas et al. (30) reported a decline from 3.8 µg.dL^−1^ in Phase 1 (2009–2013) including private-school children, to 1.3 µg.dL^−1^ in Phase 2 (2015–2019), including both private and public schools, reflecting environmental policies implemented in the early 2000s, particularly the elimination of leaded gasoline.

## 4 Discussion

The scarcity of studies on lead exposure in LAC persists. As shown by Olympio et al. (39) and Pereira et al. (40), most investigations remain confined to small “hot spot” samples, underscoring the absence of biomonitoring programs in low- and middle-income countries. Lead exposure continues to be a serious public health issue, particularly among socially vulnerable populations.

### 4.1 Effects during pregnancy, childhood and beyond

Lead exposure during pregnancy is a critical threat to fetal development (4, 22). It has been linked to preterm birth and gestational hypertension, which can progress to preeclampsia, a life-threatening complication for both mother-baby. Lead crosses the placenta and maternal and cord blood levels are strongly correlated (4). Fetal exposure thus impairs central nervous system development and has been associated with low birth weight, intrauterine growth restriction, prematurity, and spontaneous abortion (12, 38).

Evidence from previous studies confirms widespread lead exposure during pregnancy. Assis Araujo et al. (41) detected lead in 100% of Brazilian samples, with geometric means of 3.74 µg.dL^−^^1^ in maternal blood and 3.85 µg.dL^−^^1^ in umbilical cord blood. Similarly, in Colombia, Carranza-Lopez et al. (42) reported higher BLL in preterm infants than in full-term infants. The findings of this review, showing lead in cord and newborn blood, further indicate that lead toxicity begins *in utero*. This aligns with evidence from the US, where Perkins et al. (43) found that pregnant women exposed to lead, even at low BLL (mean 1.2 ± 0.59 µg.dL^−^^1^), had an increased risk of preterm birth. Similarly, in Mexico, studies indicate that prenatal lead exposure, especially in late pregnancy, represents a critical window for long-term developmental outcomes, whereas postnatal BLL show limited or inconsistent associations across studies (33, 35), underscoring the central role of prenatal exposure and early biological programming in later health and behavior (33–35).

A longitudinal study from Mexico linked prenatal and early-childhood lead exposure with delayed pubertal maturation in girls, including later secondary sexual development and menarche (44). Regional reviews show cumulative exposure in vulnerable groups, particularly women of reproductive age, but longitudinal and large-scale data on adult reproductive health, especially male fertility, remain limited.

After birth, lead exposure may amplify effects initiated during pregnancy, with childhood exposure linked to increased aggression, executive function deficits, antisocial behavior, delinquency, and violent conduct, extending to social and public safety impacts (17, 45–47). Biomarkers of cumulative exposure support associations between chronic lead exposure and antisocial behavioral outcomes (46). Together, these findings indicate that higher BLL are associated with adverse neurobehavioral and social consequences across the life course (39).

Lead exposure risk is elevated near contaminated sites linked to mining, metal and battery recycling, heavy traffic, and aging infrastructure, with exposure occurring through air, soil, dust, water, food, and lead-based paints (10, 37, 48). Lead-based paints remain a major source, so the WHO, through the Lead Paint Alliance, recommends a maximum lead content of 90 mg.kg^−1^ in paints and coatings (10). In daycare centers built before the 1940s, high environmental lead levels, higher BLL in children, and increased exposure associated with prolonged daily attendance have been observed (49, 50).

Most articles focused on environmental risk hotspots (27), underscoring persistent exposures and socio-environmental inequalities. In these regions, lead exposure prevalence is observed among children in peripheral urban areas and high-risk neighborhoods, predominantly low- or lower-middle-income, as well as among children in rural areas or experiencing poverty and malnutrition (16, 28). This pattern also occurs in women of reproductive age, with chronic exposure due to poor housing and proximity to contamination sources such as waste sites, informal industries, and home-based jewelry production (51), leading to lead accumulation in maternal bone (8).

Food insecurity is associated with increased lead absorption, as iron- and calcium-deficient diets raise BLL due to competition for intestinal absorption sites (44, 52, 53). During periods of high physiological calcium demand, such as pregnancy and lactation, intensified bone resorption can also raise BLL, as lead stored in bone is mobilized into the bloodstream (54). This may harm both mothers and breastfed children, as shown by Winiarska-Mieczan (55), who demonstrated that maternal exposure to environmental pollution and inadequate diet influence lead levels in breast milk.

Childhood lead exposure in LAC remains a public health concern, with elevated BLLs reported in diverse settings, including Mexico, where 17.4% of children aged 1–4 years had BLL ≥5.0 µg·dL^−^^1^ (15). and Colombia, where informal activities such as domestic battery recycling and lead-based fishing weight production have been associated with BLLs reaching 21.0 µg·dL^−^^1^ in children (42), The continued use of the former 5.0 µg·dL^−^^1^ reference value in several studies, despite the updated CDC benchmark of 3.5 µg·dL^−^^1^, may contribute to underestimation and underreporting of exposure.

For comparison, between 2017 and 2020, the median BLL among US children aged 1–17 years was 0.4 µg.dL^−1^ (56). In Canada, the geometric mean BLL for children aged 3–5 years during 2018–2019 was 0.50 µg.dL^−1^ (57). A study conducted in Germany, Belgium, Spain, and the Czech Republic found average BLL of 1.83 µg.dL^−1^ in children under 13 between 2003 and 2019 (58). In contrast, BLLs reported in some LAC country-specific studies, such as 1.7 µg.dL^−1^ in Jamaica (37) and 7.34 µg.dL^−1^ in Mexico (15), illustrate variability across settings and global disparities in lead exposure due to differences in environmental regulation, socioeconomic conditions, and exposure sources.

### 4.2 Sources, surveillance and prevention of lead exposure

Lead is a widespread contaminant, and identifying its sources is crucial for public health protection. WHO (8) and UNICEF (59) reports highlight lead-glazed ceramics as major exposure sources. This issue is reinforced by Pereira et al. (60), who identified studies reporting high lead and cadmium concentrations in plastic household utensils intended for children.

In recent years, regulatory advances, increased public awareness, and the gradual replacement of traditional sources have contributed to reduced lead exposure, indicating regional progress (17, 39, 40). While Mexico (12) has reported biomonitoring initiatives among pregnant women and children, many countries lack comparable efforts, leading to underreporting and hindering maternal–child health policies. Different reference values for pregnant women (5 µg·dL^−^^1^) and children (3.5 µg·dL^−^^1^) require careful interpretation. As discussed earlier, strong correlations between maternal and umbilical cord BLL indicate fetal exposure mirrors maternal burden. With no safe lead level, even asymptomatic maternal BLLs may affect fetal development, underscoring the need for pregnancy monitoring and source mitigation.

The uneven geographic distribution of studies likely reflects heterogeneous exposure contexts across ALC. In Mexico, this concentration may be partly explained by recent national policy initiatives, such as the approval in November 2019 of the *Programa de Acción de Aplicación Inmediata para el Control de la Exposición a Plomo*, which established coordinated actions for lead exposure control, including BLL surveillance. In Uruguay, the later phase-out of leaded gasoline may also have contributed to increased research attention. Longitudinal studies, such as that by Ríos-Sánchez et al. (23), indicate that although environmental remediation can reduce BLL, long-term monitoring in industrial areas often finds concentrations above reference values underscoring the need for sustained surveillance.

The data in this mini-review support routine pediatric assessment by reinforcing that the clinical effects of low-dose lead exposure are often nonspecific and may go unrecognized. Clinical manifestations include neurological symptoms (irritability, attention deficit, hyperactivity, learning difficulties), gastrointestinal complaints (abdominal pain, metallic taste), and growth or developmental delay, such as low birth weight.

Environmental risk factors should be assessed, including old housing with lead-based paint, glazed ceramics, electronic waste, and family or occupational exposures, along with the need for stricter monitoring of plastic utensils and toys containing lead pigments to meet safety standards. Training Community Health and Environmental Protection Agents to identify indoor lead sources, such as peeling paint and gates painted with red lead primer, improves protection and promotes safer environments. Educating health and education workers in environmental and reproductive health further strengthens lead prevention efforts. Adding blood lead testing to routine prenatal and early childhood visits enables early detection and timely intervention. Screening should start at the first prenatal visit using a risk questionnaire, and test when risk factors or uncertainty exist. Regulations should eliminate key sources, including lead-glazed ceramics, lead-based paints, and unsafe e-waste practices. Prevention should focus on proactive, socio-environmentally informed screening rather than waiting for symptoms, which may arise after irreversible harm. Childhood lead exposure must be assessed because early exposure is linked to later health effects, including during pregnancy and adulthood, as shown in the Kosovo cohort (61).

Substantial heterogeneity was observed across studies in exposure assessment, outcome definitions, and contextual factors (nutrition, socioeconomic vulnerability, co-exposures), limiting direct comparisons.

In the Caribbean, only a limited number of eligible studies were identified during the defined study period (2022–2026), with a single investigation conducted in Jamaica (37). Countries such as Guyana, Cuba, Trinidad and Tobago, and the Dominican Republic lack recent BLL data, creating a major gap in assessing island exposure trends. An important exception is Suriname's ongoing CCREOH cohort (62), which evaluates chemical and non-chemical stressors together. Its findings highlight single-contaminant approach limitations and the need to assess mixtures, as co-exposure to lead, arsenic, and mercury is common and increases cumulative risk. This framework has implications for LAC environmental health surveillance, population risk assessment, and preventive strategies (62, 63). Structured biomonitoring programs can thus support country-specific policies to reduce harmful metal exposures.

Future research should clarify key drivers of lead burden (socioeconomic vulnerability, nutrition, cumulative exposure, and differences in exposure sources) while strengthening longitudinal studies, improving exposure measurement, and broadening assessment of reproductive, developmental, and life-course outcomes to inform prevention, surveillance, and policy in LAC. Several studies in the LAC region have examined prenatal lead exposure and perinatal outcomes, highlighting growing concern about early-life exposure in the region and the need to link prenatal findings with later child health research. Reported associations with adverse birth outcomes, including low birth weight and preterm birth, further reinforce the public health relevance of prenatal lead exposure in these settings (64–66). Current evidence focuses mainly on prenatal exposure and early childhood effects, whereas reproductive outcomes after early-life exposure remain understudied in the region.

## References

[1] Borja-Aburto VH Hertz-Picciotto I Lopez MR Farias P Rios C Blanco J. Blood lead levels measured prospectively and risk of spontaneous abortion. Am J Epidemiol. (1999) 150(6):590–7. 10.1093/oxfordjournals.aje.a010057 10489998

[2] Guerra-Tamayo JL Hernández-Cadena L Téllez-Rojo MM Mercado-García ADS Solano-González M Hernández-Avila M Exposición al plomo y su relación con El Tiempo requerido para embarazo. Salud Pública México. (2003) 45:189–95. 10.1590/S0036-36342003000800004 14750500

[3] Bayat F Amir Ali Akbari S Dabirioskoei A Nasiri M Mellati A. The relationship between blood lead level and preeclampsia. Electron Physician. (2016) 8(12):3450–5. 10.19082/3450 28163864 PMC5279982

[4] Yang YH Liou SH Yang CY Sung FC Wu CC Wu TN. Increased blood lead concentration during menstruation in teen female students. Sci Total Environ. (2007) 382(2–3):224–7. 10.1016/j.scitotenv.2007.04.021 17543370

[5] Alexander BH Checkoway H Van Netten C Muller CH Ewers TG Kaufman JD Semen quality of men employed at a lead smelter. Occup Environ Med. (1996) 53(6):411–6. 10.1136/oem.53.6.411 8758037 PMC1128498

[6] Lancranjan I Popescu HI Găvănescu O Klepsch I Serbănescu M. Reproductive ability of workmen occupationally exposed to lead. Arch Environ Health. (1975) 30(8):396–401. 10.1080/00039896.1975.10666733 1155972

[7] World Health Organization. Reproductive Health Indicators: Guidelines for Their Generation, Interpretation and Analysis for Global Monitoring. Geneva: World Health Organization (2006). p. 63.

[8] Ettinger AS Wengrovitz AG, editors. Guidelines for the identification and management of lead exposure in pregnant and lactating women. Atlanta (GA): U.S. Department of Health and Human Services, Centers for Disease Control and Prevention, National Center for Environmental Health/Agency for Toxic Substances and Disease Registry (2010).

[9] Council on Environmental Health, Lanphear BP Lowry JA Ahdoot S Baum CR Bernstein AS Prevention of childhood lead toxicity. Pediatrics. (2016) 138(1):e20161493. 10.1542/peds.2016-1493 27325637

[10] Global Elimination of Lead Paint: Why and How Countries Should Take Action. Technical Brief. 1st Ed Geneva: World Health Organization (2020). p. 1.

[11] Centers for Disease Control and Prevention. CDC updates blood lead reference value (2025). Available online at: https://www.cdc.gov/lead-prevention/php/news-features/updates-blood-lead-reference-value.html (Accessed February 14, 2026).

[12] Flannery BM Dolan LC Hoffman-Pennesi D Gavelek A Jones OE Kanwal R U.S. Food and drug Administration's Interim reference levels for dietary lead exposure in children and women of childbearing age. Regul Toxicol Pharmacol. (2020) 110:104516. 10.1016/j.yrtph.2019.104516 31707132

[13] Bah HAF Dos Anjos ALS Gomes-Júnior EA Bandeira MJ De Carvalho CF Dos Santos NR Delta-Aminolevulinic acid dehydratase, low blood lead levels, social factors, and intellectual function in an afro-Brazilian children community. Biol Trace Elem Res. (2022) 200(2):447–57. 10.1007/s12011-021-02656-8 33723800

[14] Landrigan PJ Fuller R Acosta NJR Adeyi O Arnold R Basu N The lancet commission on pollution and health. Lancet. (2018) 391(10119):462–512. 10.1016/S0140-6736(17)32345-0 29056410

[15] Figueroa JL Rodríguez-Atristain A Bautista-Arredondo LF Treviño CL Martínez MR Valdivia BT Loss of cognitive function in Mexican children due to lead exposure and the associated economic costs. Environ Res. (2024) 263:120013. 10.1016/j.envres.2024.120013 39284488

[16] Agudelo N Cuadro A Barg G Queirolo EI Mañay N Kordas K. Blood lead levels and math learning in first year of school: an association for concern. Environ Res. (2024) 246:118091. 10.1016/j.envres.2023.118091 38215927 PMC10947836

[17] Needleman H. Lead poisoning. Annu Rev Med. (2004) 55(1):209–22. 10.1146/annurev.med.55.091902.103653 14746518

[18] Haddaway NR Page MJ Pritchard CC McGuinness LA. PRISMA2020: an R package and shiny app for producing PRISMA 2020-compliant flow diagrams, with interactivity for optimised digital transparency and open synthesis. Campbell Syst Rev. (2022) 18(2):e1230. 10.1002/cl2.1230 36911350 PMC8958186

[19] Ouzzani M Hammady H Fedorowicz Z Elmagarmid A. Rayyan—a web and mobile app for systematic reviews. Syst Rev. (2016) 5(1):210. 10.1186/s13643-016-0384-4 27919275 PMC5139140

[20] Córdoba-Gamboa L Vázquez-Salas RA Romero-Martínez M Cantoral A Riojas-Rodríguez H Bautista-Arredondo S Lead exposure can affect early childhood development and could be aggravated by stunted growth: perspectives from Mexico. Int J Environ Res Public Health. (2023) 20(6):5174. 10.3390/ijerph20065174 36982080 PMC10049063

[21] Téllez-Rojo MM Bautista Arreondo LF Cantoral-Preciado A Peralta N Figueroa JL Trejo-Valdivia B. Intoxicación por plomo en población pediátrica. Salud Pública México. (2024) 66(4):467–76. 10.21149/15840 39977098

[22] Bautista-Arredondo LF Trejo-Valdivia B Estrada-Sánchez D Tamayo-Ortiz M Cantoral A Figueroa JL Intoxicación infantil por plomo en méxico: otras fuentes de exposición más allá del barro vidriado (ensanut 2022). Salud Pública México. (2023) 65:s197–203. 10.21149/14798 38060959

[23] Ríos-Sánchez E Rubio-Andrade M García-Vargas GG. Vigilancia epidemiológica en población infantil expuesta a plomo en coahuila. México. Salud Pública México. (2025) 67(5):485–93. 10.21149/16774 41150988

[24] Agudelo N Cuadro A Barg G Queirolo EI Mañay N Kordas K. The relationship between lead levels and Reading acquisition in Spanish speakers, evidence from Uruguayan schoolers. NeuroToxicology. (2024) 105:272–9. 10.1016/j.neuro.2024.10.011 39532269 PMC11645207

[25] Linares AM Thaxton-Wiggins A Unrine JM. Concentrations of lead and arsenic in mother’s milk and children’s blood in Peruvian breastfeeding dyads. J Hum Lact. (2024) 40(1):69–79. 10.1177/08903344231212430 38084709 PMC10984648

[26] Medel-Jara P Gejman C Chavez B Saavedra M Paredes F Valenzuela A Metal exposure in Arica, Chile: examining toxic elements. Rev Méd Chile. (2023) 151(4):420–7. 10.4067/s0034-98872023000400420 38687516

[27] Disalvo L Cassain V Fasano MV Zar G Varea A Virgolini MB. Environmental exposure to lead and oxidative stress biomarkers among healthy children in La Plata, Argentina. Arch Argent Pediatr. (2022) 120(3):174–9. 10.5546/aap.2022.eng.174 35533119

[28] Disalvo L Varea A Matamoros N Sala M Fasano MV González HF. Blood lead levels and their association with iron deficiency and Anemia in children. Biol Trace Elem Res. (2025) 203(1):69–75. 10.1007/s12011-024-04163-y 38568334

[29] Barg G Menéndez JA Friedl JA Hoyos S Queirolo EI Mañay N Lead exposure, peripheral neurotransmitter levels and cognitive control: a neurobehavioral study in children from Montevideo, Uruguay. NeuroToxicology. (2025) 108:159–68. 10.1016/j.neuro.2025.03.009 40189059

[30] Kordas K Thomas M Millen AE Queirolo EI Mañay N Peregalli F Diet quality and blood lead levels in Uruguayan first graders. Sci Total Environ. (2024) 954:176545. 10.1016/j.scitotenv.2024.176545 39332730 PMC13037589

[31] Merced-Nieves FM Chelonis J Pantic I Schnass L Téllez-Rojo MM Braun JM Sexually dimorphic associations between prenatal blood lead exposure and performance on a behavioral testing battery in children. Neurotoxicol Teratol. (2022) 90:107075. 10.1016/j.ntt.2022.107075 35108597 PMC8957713

[32] Liu SH Chen Y Bellinger D De Water E Horton M Téllez-Rojo MM Pre-natal and early life lead exposure and childhood inhibitory control: an item response theory approach to improve measurement precision of inhibitory control. Environ Health. (2024) 23(1):71. 10.1186/s12940-023-01015-5 39232724 PMC11375946

[33] Svensson K Lane JM Chelonis JJ Gennings C Bellinger DC Hernández-Chávez C Developmental Pb exposure increases rate of forgetting on a delayed matching-to-sample task among Mexican children. Sci Adv. (2025) 11(11):eadq4495. 10.1126/sciadv.adq4495 40632840 PMC12239937

[34] Tagelsir AA Hector E Urena-Cirett J Mercado-Garcia A Cantoral A Hu H Early lead exposure is associated with molar hypomineralization. Pediatr Dent. (2023) 45(5):427–33. PMID: 37904269 PMC10936227

[35] Reyes Sánchez JD Trejo-Valdivia B Schnaas L Osorio-Valencia E Lamadrid-Figueroa H Bautista-Arredondo LF Early life exposure to lead and its association with parent-reported aggression and conduct problems during childhood and adolescence. NeuroToxicology. (2022) 93:265–71. 10.1016/j.neuro.2022.10.008 36252845

[36] Lane JM Liu SH Midya V Alcala CS Eggers S Svensson K Childhood pb-induced cognitive dysfunction: structural equation modeling of hot and cold executive functions. J Expo Sci Environ Epidemiol. (2025) 35(5):715–24. 10.1038/s41370-025-00761-7 40033031 PMC12354184

[37] Rahbar MH Samms-Vaughan M Kim S Saroukhani S Bressler J Hessabi M Detoxification role of metabolic glutathione S-transferase (GST) genes in blood lead concentrations of Jamaican children with and without autism Spectrum disorder. Genes (Basel). (2022) 13(6):975. 10.3390/genes13060975 35741737 PMC9222697

[38] Queirolo EI Kordas K Martínez G Ahmed Z Barg G Mañay N. Secular trends in blood lead concentrations of school-age children in Montevideo, Uruguay from 2009 to 2019. Environ Pollut. (2024) 343:123160. 10.1016/j.envpol.2023.123160 38104764 PMC10922799

[39] Olympio KPK Gonçalves CG Salles FJ Ferreira APSDS Soares AS Buzalaf MAR What are the blood lead levels of children living in Latin America and the Caribbean? Environ Int. (2017) 101:46–58. 10.1016/j.envint.2016.12.022 28159393

[40] Pereira EC Piai KDA Salles FJ Silva ASD Olympio KPK. A comprehensive analysis of children’s blood lead levels in Latin America and the Caribbean over the last eight years: progress and recommendations. Sci Total Environ. (2024) 928:172372. 10.1016/j.scitotenv.2024.172372 38604359

[41] De Assis Araujo MS Figueiredo ND Camara VM Froes Asmus CIR. Maternal-child exposure to metals during pregnancy in rio de janeiro city, Brazil: the rio birth cohort study of environmental exposure and childhood development (PIPA project). Environ Res. (2020) 183:109155. 10.1016/j.envres.2020.109155 32006767

[42] Carranza-Lopez L Alvarez-Ortega N Caballero-Gallardo K Gonzalez-Montes A Olivero-Verbel J. Biomonitoring of lead exposure in children from two fishing communities at northern Colombia. Biol Trace Elem Res. (2021) 199(3):850–60. 10.1007/s12011-020-02207-7 32488615

[43] Perkins M Wright RO Amarasiriwardena CJ Jayawardene I Rifas-Shiman SL Oken E. Very low maternal lead level in pregnancy and birth outcomes in an eastern Massachusetts population. Ann Epidemiol. (2014) 24(12):915–9. 10.1016/j.annepidem.2014.09.007 25444892 PMC4254591

[44] Hegazy AA Zaher MM Abd El-Hafez MA Morsy AA Saleh RA. Relation between anemia and blood levels of lead, copper, zinc and iron among children. BMC Res Notes. (2010) 3(1):133. 10.1186/1756-0500-3-133 20459857 PMC2887903

[45] Dietrich KN Douglas RM Succop PA Berger OG Bornschein RL. Early exposure to lead and juvenile delinquency. Neurotoxicol Teratol. (2001) 23(6):511–8. 10.1016/S0892-0362(01)00184-2 11792521

[46] Olympio KPK Oliveira PV Naozuka J Cardoso MRA Marques AF Günther WMR Surface dental enamel lead levels and antisocial behavior in Brazilian adolescents. Neurotoxicol Teratol. (2010) 32(2):273–9. 10.1016/j.ntt.2009.12.003 20005947

[47] Obamuyide H Blose N Kredo T Matzopoulos R. Early life lead exposure as a risk factor for aggressive and violent behaviour in young adults: a systematic review. Aggress Violent Behav. (2025) 85:102090. 10.1016/j.avb.2025.102090

[48] Piai KDA Olympio KPK. Children’s blood lead levels in Latin America and the Caribbean—recommendations to combat this well-known persistent public health problem. Curr Opin Environ Sci Health. (2023) 32:100454. 10.1016/j.coesh.2023.100454

[49] Olympio KPK Silva JPDR Silva ASD Souza VCDO Buzalaf MAR Barbosa F Jr Blood lead and cadmium levels in preschool children and associated risk factors in São Paulo, Brazil. Environ Pollut. (2018) 240:831–8. 10.1016/j.envpol.2018.04.124 29783200

[50] Leroux IN Ferreira APSDS Silva JPDR Bezerra FF Da Silva FF Salles FJ Lead exposure from households and school settings: influence of diet on blood lead levels. Environ Sci Pollut Res. (2018) 25(31):31535–42. 10.1007/s11356-018-3114-8 30203353

[51] Salles FJ Atilola G Frydas I Schultz DR Papaioannou N Rogero MM Effects of minimal arsenic, lead, and cadmium exposure on biological pathways in Brazilian informal workers welding fashion jewelry. J Trace Elem Med Biol. (2025) 89:127660. 10.1016/j.jtemb.2025.127660 40300411

[52] Bradman A Eskenazi B Sutton P Athanasoulis M Goldman LR. Iron deficiency associated with higher blood lead in children living in contaminated environments. Environ Health Perspect. (2001) 109(10):1079–84. 10.1289/ehp.011091079 11675273 PMC1242086

[53] Wright RO Tsaih SW Schwartz J Wright RJ Hu H. Association between iron deficiency and blood lead level in a longitudinal analysis of children followed in an urban primary care clinic. J Pediatr. (2003) 142(1):9–14. 10.1067/mpd.2003.mpd0344 12520247

[54] Ettinger AS Hu H Hernandez-Avila M. Dietary calcium supplementation to lower blood lead levels in pregnancy and lactation. J Nutr Biochem. (2007) 18(3):172–8. 10.1016/j.jnutbio.2006.12.007 17296490 PMC2566736

[55] Winiarska-Mieczan A. Cadmium, lead, copper and zinc in breast milk in Poland. Biol Trace Elem Res. (2014) 157(1):36–44. 10.1007/s12011-013-9870-x 24338444 PMC3895183

[56] EPA. Biomonitoring—lead. Available online at: https://www.epa.gov/americaschildrenenvironment/biomonitoring-lead (Accessed October 31, 2025).

[57] Health Canada. Sixth report on human biomonitoring of environmental chemicals in Canada—page 2 (2021). Available online at: https://www.canada.ca/en/health-canada/services/environmental-workplace-health/reports-publications/environmental-contaminants/sixth-report-human-biomonitoring/page-2.html (Accessed November 2, 2025).

[58] Salamanca-Fernández E Peinado FM Esteban-López M Puklova V Poyatos RM Govarts E Sociodemographic determinants and temporal variability of blood lead levels (2003–2019) in a pooled analysis of nine studies in four European countries. Sci Rep. (2025) 15(1):34562. 10.1038/s41598-025-17943-w 41044099 PMC12494996

[59] UNICEF; Pure Earth. The toxic truth: children’s exposure to lead pollution undermines a generation of future potential (2020). Available online at: https://www.unicef.org/reports/toxic-truth-childrens-exposure-lead-pollution-2020 (Accessed October 31, 2025).

[60] Pereira EC Leroux IN Luz MS Batista BL Olympio KPK. Study of controlled migration of cadmium and lead into foods from plastic utensils for children. Environ Sci Pollut Res. (2022) 29(35):52833–43. 10.1007/s11356-022-19433-2 35275370

[61] Camaj PR Graziano JH Preteni E Popovac D Loiacono N Balac O Long-Term effects of environmental lead on erythropoietin production in young adults: a follow-up study of a prospective cohort in Kosovo. J Environ Public Health. (2020) 2020:1–9. 10.1155/2020/3646252 PMC7785392 33456476

[62] Zijlmans W Wickliffe J Hindori-Mohangoo A MacDonald-Ottevanger S Ouboter P Landburg G. Caribbean Consortium for Research in Environmental and Occupational Health (CCREOH) cohort study: influences of complex environmental exposures on maternal and child health in Suriname. BMJ Open. (2020) 10:e034702. 10.1136/bmjopen-2019-034702 32928846 PMC7488800

[63] Baldewsingh GK Wickliffe JK Van Eer ED Shankar A Hindori-Mohangoo AD Harville EW Prenatal mercury exposure in pregnant women from Suriname’s Interior and its effects on birth outcomes. Int J Environ Res Public Health. (2020) 17(11):4032. 10.3390/ijerph17114032 32517037 PMC7312160

[64] Berky AJ Weinhouse C Vissoci J Rivera N Ortiz EJ Navio S In utero exposure to metals and birth outcomes in an artisanal and small-scale gold mining birth cohort in Madre de Dios, Peru. Environ Health Perspect. (2023) 131(9):097008. 10.1289/EHP10557 37747404 PMC10519195

[65] Sewberath Misser VH Hindori-Mohangoo AD Shankar A Lichtveld M Wickliffe J Mans DRA. Possible risk factors and their potential associations with combined heavy metal exposures in pregnant women in the republic of Suriname. Ann Glob Health. (2024) 90(1):30. 10.5334/aogh.4402 38618276 PMC11011959

[66] Alegría-Torres JA Rocha-Amador DO Pérez-Rodríguez RY Rodríguez-Felipe VM Cauich-Díaz M Ponce-Noyola P Pilot monitoring of lead in umbilical cord blood of newborns associated with the use of glazed ceramics from guanajuato, Mexico. Biol Trace Elem Res. (2024) 202(6):2403–9. 10.1007/s12011-023-03843-5 37702961

