# Supplementary material for: Lead exposure across early life in Latin America and the Caribbean: prevention strategies and reproductive health considerations
Source: Front Reprod Health. 2026 Mar 18;8:1761778. doi: 10.3389/frph.2026.1761778 (PMC13038884; doi:10.3389/frph.2026.1761778)
Supplement: Supplementary file 1 [file Table1.docx]

**Supplementary Table 1:**

Search strategy adopted for each scientific database.

| **Scientific databases and criteria** | **Search parameters** |
| --- | --- |
| PubMed | (("Lead"[Title/Abstract] OR "Plomo"[Title/Abstract] OR "Chumbo"[Title/Abstract]) AND "Blood"[Title/Abstract] AND ("Latin America"[MeSH Terms] OR "South America"[MeSH Terms] OR "Central America"[MeSH Terms] OR "Mexico"[MeSH Terms] OR "Caribbean Region"[MeSH Terms]) AND ("infant"[MeSH Terms] OR "child"[MeSH Terms] OR "adolescent"[MeSH Terms] OR ("Pregnancy"[Title/Abstract] OR "prenatal"[Title/Abstract] OR "maternal"[Title/Abstract] OR "Maternal Exposure"[MeSH Terms] OR "Pregnancy"[MeSH Terms] OR "Prenatal Exposure Delayed Effects"[MeSH Terms] OR "Prenatal Care"[MeSH Terms])) AND 2022/01/01:2026/01/29[Date - Publication]) |
| Web of Science | (lead OR plomo OR chumbo) (Topic) AND blood (Topic) AND (child* OR infant* OR adolescen* OR pediatric* OR paediatric*) OR (pregnancy OR prenatal OR maternal OR "Prenatal Exposure Delayed Effects" OR "Prenatal Care") (Topic) AND ("Latin America" OR "South America" OR "Central America" OR Mexico OR Caribbean OR "Caribbean Region") (Topic) and Article (Document Types) and 2022 or 2023 or 2024 or 2025 (Publication Years) |
| Lilacs | (chumbo OR plomo OR lead) AND (sangue OR sangre OR blood) AND ((lactente OR Lactante OR preescolar OR crianca OR adolescente OR niño) OR (gravidez OR Pregnancy OR Embarazo OR prenatal OR materno OR "Maternal Exposure" OR "Pregnancy" OR "Prenatal Exposure Delayed Effects" OR "Prenatal Care")) |
| Inclusion criteria | Studies in English, Portuguese, and Spanish were included. Only studies reporting blood lead levels in children and mother-child pairs were included. |
| Exclusion criteria | Studies were excluded if they focused exclusively on adults or pregnant women without presenting child-specific BLL data, or if results for children were not reported separately. Articles using biological matrices other than blood (e.g., hair, nails, teeth) or relying solely on umbilical cord blood were excluded. Reviews, editorials, letters, commentaries, and studies based exclusively on secondary data were also excluded, as were studies conducted outside Latin America and the Caribbean or published outside the defined time frame. |
|  |  |
