# Supplementary figures and images for: Lead exposure across early life in Latin America and the Caribbean: prevention strategies and reproductive health considerations
Source: Front Reprod Health. 2026 Mar 18;8:1761778. doi: 10.3389/frph.2026.1761778 (PMC13038884; doi:10.3389/frph.2026.1761778)

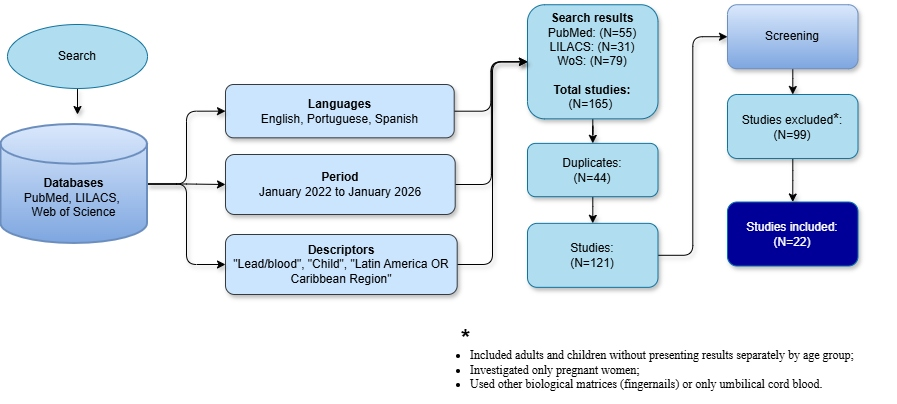

Supplement: Supplementary file 2 [file Image1.tiff]
